# Global lncRNA expression profiles in medulloblastoma reveal crucial lncRNA-oncogene interactions in Sonic hedgehog and Group 4

**DOI:** 10.1093/noajnl/vdaf194

**Published:** 2025-09-04

**Authors:** Iván Martínez de Estíbariz, Unai Illarregi, Daniel Sinnett, Angela Gutiérrez-Camino, Elixabet Lopez-Lopez, Laura Zaldumbide, Laura Zaldumbide, Miguel García-Ariza, Lide Alaña, Luis F Callado, Nerea Bilbao-Aldaiturriaga, Idoia Martin-Guerrero

**Affiliations:** Department of Genetics, Physical Anthropology and Animal Physiology, Faculty of Science and Technology, University of the Basque Country (UPV/EHU), Leioa, Spain; Department of Genetics, Physical Anthropology and Animal Physiology, Faculty of Science and Technology, University of the Basque Country (UPV/EHU), Leioa, Spain; Department of Pediatrics, Faculty of Medicine, University of Montreal, Montreal, Quebec, Canada; Research Center, CHU Sainte-Justine, Montreal, Quebec, Canada; Pediatric Oncology Group, BioBizkaia Health Research Institute, Barakaldo, Spain; Research Center, CHU Sainte-Justine, Montreal, Quebec, Canada; Department of Biochemistry and Molecular Biology, Faculty of Science and Technology, University of the Basque Country (UPV/EHU), Leioa, Spain; Pediatric Oncology Group, BioBizkaia Health Research Institute, Barakaldo, Spain; Department of Pathology, Cruces University Hospital, Barakaldo, Spain; Department of Pathology, Cruces University Hospital, Barakaldo, Spain; Pediatric Oncology Group, BioBizkaia Health Research Institute, Barakaldo, Spain; Pediatric Oncology Group, BioBizkaia Health Research Institute, Barakaldo, Spain; Department of Pharmacology, University of the Basque Country (UPV/EHU), Leioa, Spain; Centro de Investigación Biomédica en Red de Salud Mental, CIBERSAM, ISCIII, Madrid, Spain; Pediatric Oncology Group, BioBizkaia Health Research Institute, Barakaldo, Spain; Pediatric Oncology Group, BioBizkaia Health Research Institute, Barakaldo, Spain; Department of Genetics, Physical Anthropology and Animal Physiology, Faculty of Science and Technology, University of the Basque Country (UPV/EHU), Leioa, Spain

**Keywords:** cancer biomarkers, gene expression and interaction, long noncoding RNAs, medulloblastoma, therapeutic targets

## Abstract

**Background:**

Advances in multi-omic studies have improved medulloblastoma (MB) characterization, yet novel molecular biomarkers are needed to refine tumor biology and therapeutic strategies. Current profiling mainly targets the protein-coding genome, while the potential of noncoding regions remains unexplored. This study aims to identify long noncoding RNAs (lncRNAs), emerging as crucial regulators in MB, as potential key biomarkers specific to molecular group, enhancing understanding of MB’s genomic landscape.

**Methods:**

RNA-seq data from 54 Spanish MB patients (C1) and 207 public samples (C2) were analyzed to profile lncRNAs. Expression and Weighted Gene Coexpression Network (WGCNA) analyses were performed to identify lncRNA-oncogene interactions. Group-specific interactions were examined to infer their role in MB pathogenesis and highlight potential lncRNA involvement in disease mechanisms.

**Results:**

LncRNA expression profiles identified 4 clusters corresponding to the MB molecular groups, confirming their potential as biomarkers. Expression and WGCNA analyses revealed group-specific lncRNAs for Sonic hedgehog (SHH), Group 3 (Gr3), and Group 4 (Gr4) MB. *Lnc-SMARCA2* was exclusively upregulated in SHH MB, and associated with *ATOH1* and *PDLIM3*, key cilium regulators of this group’s cell of origin. In Gr4 MB, *MGC32805* and *LOC107986446* were upregulated and linked to *SNCAIP*, potentially influencing *PRDM6* activation via enhancer hijacking. Additionally, a 5-lncRNA signature linked to phototransduction was exclusive to Gr3, offering insights into its lineage switch and molecular regulation.

**Conclusions:**

*Lnc-SMARCA2* and, *MGC32805* and *LOC107986446*, are exclusively deregulated in SHH and Gr4 MB, respectively, and directly associated with group-specific MB oncogenes, representing promising novel biomarkers and therapeutic targets in MB.

## Graphical Abstract

**Graphical Abstract:**
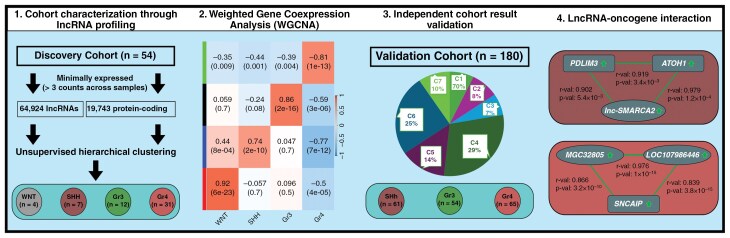

**Key Points:**

- Expression analysis of lncRNAs highlights their potential as biomarkers in MB.
- *Lnc-SMARCA2* may be a novel biomarker and potential therapeutic target in SHH MB.
- *MGC32805* and *LOC107986446* may intercede *PRDM6* activation for Gr4 MB development.

**Importance of the Study:**

SHH, Gr3, and Gr4 molecular groups are associated with poor clinical outcomes due to the challenge characterizing patients within these groups. To address this, we identified 3 lncRNAs correlated with driver MB genes through an in silico analysis of 54 (C1) and 207 MBs (C2). In SHH patients, *lnc-SMARCA2* was significantly correlated with *PDLIM3* and *ATOH1*, involved in SHH development via aberrant ciliogenesis in granule neuron progenitor cells. In Gr4 MB, *SNCAIP* exhibited strong interactions with *MGC32805* and *LOC107986446* lncRNAs, suggesting a link between their aberrant expression and *SNCAIP* duplication, leading to *PRDM6* activation and Gr4 MB development. Additionally, a 5-lncRNA signature implicated in the transition between unipolar brush cells and photoreceptor lineage in Gr3 MB was identified. These findings underscore the critical role of lncRNAs in MB, suggesting *lnc-SMARCA2*, *MGC32805*, and *LOC107986446* as candidate biomarkers and potential therapeutic targets in SHH and Gr4 MB.

Medulloblastoma (MB) is an aggressive pediatric brain tumor originating in the cerebellum or posterior fossa.^1^ With an annual incidence of 1.8 cases per million population, MB is one of the most common CNS malignancies (WHO Grade IV) in childhood, accounting for ~20% of intracranial neoplasms in this age group.^2^ Medulloblastoma is a highly heterogeneous disease, characterized by distinct clinical presentations, molecular features, and survival outcomes.^3^ Advances in understanding this heterogeneity have led to improved classification and risk stratification systems,^4^ which resulted in the 2021 WHO classification of CNS tumors, delineating MB into 4 molecular groups: Wingless/INT1 (WNT)-activated MB, Sonic hedgehog (SHH)-activated MB, Group 3 (Gr3), and Group 4 (Gr4).^5^ Briefly, WNT-activated MBs comprise ~10% of all MBs and typically show excellent prognosis, with overall survival (OS) rates approaching 100%.^1,6^ Sonic hedgehog-activated MBs account for ~30% of all MB cases, being stratified based on *TP53* status into *TP53*-mutant (~15% of SHH MBs) and *TP53*-wildtype tumors, with OS rates of ~40% and ~75%, respectively.^6^ Gr3 MBs constitute ~25% of all cases and are regarded as the most aggressive molecular group, with OS rates of ~50%,^7^ while Gr4 MBs represent the largest molecular group, accounting for up to 40% of all cases, with a prognosis that varies widely from intermediate to very poor outcomes.^8^

Even if these advances have clearly changed the landscape of MB pathogenesis, still one third of high-risk patients and ~25% of low/standard-risk patients are incurable.^9^ To address these challenges, there is an urgent necessity of identifying new biomarkers that could enlighten novel approaches for targeted therapies for patients that currently lack effective characterization. Up to now, all tumor biomarkers that are utilized for MB classification have been derived from studies focusing on the protein-coding regions of the genome, with the noncoding regions largely overlooked. Recently, attention has shifted toward the noncoding RNA (ncRNA) landscape, which constitutes the predominant portion of the genome, highlighting its potential as a source for identifying novel biomarkers to enhance MB molecular profiling.

Among ncRNAs, lncRNAs represent the largest subgroup, with over 100 000 identified in the human genome.^10^ Although the functions of only a small fraction have been characterized, lncRNAs play key roles in regulating chromatin structure, transcription, or microRNA activity, making them critical factors in cancer biology.^10^ Notably, around 40% of annotated lncRNAs are exclusively expressed in the brain, where they are highly evolutionarily conserved and often located near protein-coding genes involved in neuronal differentiation and function.^11^ Furthermore, aberrant expression of specific lncRNAs has been observed across different molecular groups of MB, underscoring their potential utility as diagnostic and prognostic biomarkers, and critical role in the pathogenesis of MB.^12^ For instance, lncRNAs *HHIP-AS1* and *LOXL1-AS1* have been implicated in promoting tumorigenicity and metastasis in SHH-driven MBs, whereas *NKX2-2AS* functions as a tumor suppressor in these tumors.^13–15^ In Gr3 MB, *lnc-HLX-2-7* facilitates tumor progression, whereas in Gr4 MB, *SPRIGHTLY* drives tumor growth.^16,17^ Additionally, several lncRNAs such as *LINC-NED125*, *HOTAIR*, *CRNDE*, or *OTX2-AS1* have been identified as promoters of MB cell proliferation, metastasis, migration, or invasion, respectively, highlighting their role in tumor biology.^18–21^

In this study, we conducted a comprehensive characterization of lncRNA expression profiles in MB patients using 2 independent cohorts, highlighting the potential of lncRNAs as biomarkers in the disease. Correlation analyses were performed in both cohorts across the 4 molecular groups to identify group-specific lncRNA candidates that could act as potential therapeutic biomarkers in MB molecular groups. This approach allowed us to identify 2 pivotal lncRNAs–protein–coding gene interaction hub nodes with potential roles in the pathogenesis of SHH and Gr4 MB, identifying 3 lncRNAs which may serve as key molecular biomarkers for these groups.

## Methods

### Population of the study

This study included 2 independent cohorts: Cohort 1 (C1), used as the discovery cohort; and Cohort 2 (C2), used as an independent cohort to test the reproducibility of results. C1 included 54 pediatric patients diagnosed with MB between 2016 and 2022 from 23 different hospitals in Spain. The clinical data of this cohort included age at diagnosis, gender, clinical risk markers (residual disease and metastasis), biological risk markers (*MYC* amplification, driver mutations, predisposition syndromes, histological phenotype), and molecular group classification based on immunohistochemistry and methylation (minimal methylation classifier assay; MS-MIMIC) profiling.^22^ Additionally, independent and publicly available cohorts from the Gene Expression Omnibus repository, including GSE143940, GSE148389, GSE151519, GSE158413, GSE164677, GSE181293, and GSE243795, were used as C2 to find group-specific gene interactions and test the reproducibility of findings between cohorts. Raw FASTQ files, for a total of 207 patients, were downloaded directly from each cohort for downstream analyses. Samples missing molecular group information were excluded from the study. All demographic and clinical data available for C1 and C2 are provided in Supplementary Table 1.

### RNA-seq library preparation

RNA was extracted at diagnosis from tumor tissue obtained from cerebellum biopsies of the 54 C1 patients using the All Prep DNA/RNA Mini kit (Qiagen). The quality and integrity of the isolated RNA were assessed with the Bioanalyzer 2100 system (Agilent Technologies). Next, RNA libraries were prepared and processed with 1 µg of RNA using Illumina Stranded Total RNA Ribo-Zero Plus kit and IDT Unique Dual Indexes (Illumina), following the manufacturer’s instructions. The resulting stranded libraries were sequenced at the Integrated Centre for Pediatric Clinical Genomics, CHU Sainte-Justine Research Centre (Montreal, Canada), on the NovaSeq 6000 platform (Illumina), generating ~150 million 100 bp paired-end reads per sample.

### Preprocessing, mapping, and quantification of raw sequencing data

Raw FASTQ files were processed and trimmed with *fastp*,^23^ and quality was assessed using *FastQC*.^24^ Filtered reads were aligned against the GRCh38/hg38 reference genome with STAR aligner (v2.7.10a),^25^ and gene-level quantification was performed using featureCounts^26^ and the lncRNA knowledgebase (lncRNAKB)^27^ annotation, an enriched lncRNA GFF database comprising 73,611 human lncRNAs and 22,518 protein-coding genes. Given the technical differences likely affecting downstream correlation detectability, batch correction was performed on the raw data of C2 using the ComBat-seq method from the *sva* package.^28^ Finally, minimally expressed (>3 read counts across samples) raw read counts were normalized using the *vst* function in the *DESeq2* package (R v4.1.1/Bioconductor).^29^

### Unsupervised lncRNA-based hierarchical clustering and patient molecular group classification

Unsupervised hierarchical clustering (HCL), based on t-distributed stochastic neighbor embedding, was conducted on C1 for molecular group assignation. For this analysis, the 5000 genes with the highest variability among samples wee identified using the *var* function in R, of which 2,474 were lncRNAs. For the HCL *Rtsne* and *ggplot2* packages were used, C1 was classified into the 4 molecular groups by integrating all available clinical data with the results from the lncRNA-based HCL analysis.

### LncRNA differential expression analysis of the 4 established MB groups

Differential expression analysis (DEA) was performed using *DESeq2*, comparing each of the molecular groups against the other 3 groups. Differentially expressed lncRNAs were identified to define molecular group specific lncRNA profiles. LncRNAs with a false discovery rate (FDR) corrected *P*-value < .05 and log2FoldChange (log2FC) >|1.5| were considered as significantly deregulated. Expression profiles were first derived from C1, while C2 was used to assess minimum reproducibility.

### Weighted gene coexpression network analysis

The coexpression network of C1 was constructed using the *WGCNA* package (v1.72-1).^30^ Weighted Gene Coexpression Network (WGCNA) to the 5000 most variable genes was applied with the following parameters; a soft threshold power of 10, a minimum module size of 50 genes, and a cut height of 0.35 (Supplementary Figure 1). Correlation analyses were performed between the gene modules of the created network and specific traits (corresponding to the four molecular groups of MB). Gene modules were considered significantly correlated with traits if they exhibited *r* > |0.7| and *P*-value ≤ .01. The most significant and strongly correlated modules to each trait were selected. Finally, to assess the significance of differences in eigengene values between traits, a Wilcoxon Mann–Whitney test was performed. All the analyses were performed using R software (v4.1.1).

### Selection of the group-specific interactions between lncRNAs and protein-coding genes

Overrepresentation analysis (ORA) to validate the representativeness of the significant modules with the traits was conducted using ConsensusPathDB.^31^ Pathways were considered significantly overrepresented if they met an FDR-corrected *P*-value threshold of *q* ≤ 0.05. To infer interactions between lncRNAs and protein-coding genes specific to molecular groups, we applied Pearson’s correlation analysis. This analysis calculated correlations between differentially expressed protein-coding genes and lncRNAs within the modules of the most correlated molecular groups, constructing a group-specific gene correlation network. The WNT molecular group’s module was excluded from the analysis due to insufficient sample size for Pearson’s correlation test (*n* = 5). Once lncRNA-protein-coding gene correlations were identified in C1, Pearson’s correlation analysis with the same input was performed on C2. Only significant interactions overlapping between cohorts for each of the module-trait pairs were selected for further analyses (*r* > |0.7| and *P* < .05). These lncRNA–protein–coding gene interactions were individually assessed to infer their relationship with the disease in literature and ORA to select potentially driver gene pairs associated with each of the molecular groups.

### Identification of potential regulatory mediators of lncRNA–protein–coding gene interactions

To investigate potential regulatory mechanisms underlying the lncRNA–protein–coding gene interactions, we performed gene regulatory network (GRN) inference using the Adaptive Partitioning version of the Algorithm for the Reconstruction of Accurate Cellular Networks (ARACNe-AP).^32^ Transcription factors (TFs) were selected to study potentially mediating molecules in the observed correlations. Transcription factor annotation was retrieved from *AnimalTFDB 4.0* database.^33^ Batch-corrected and normalized gene expression profiles from both cohorts were used as input. Mutual information thresholds for SHH and Gr4 patients were estimated with the *calculateThreshold* function. The gene regulatory network was constructed by running 100 bootstrap iterations and consolidating the resulting network with the *consolidate* function. Following network construction, we specifically examined interactions involving the previously identified lncRNA–protein–coding gene pairs and their potential regulatory TFs.

## Results

### Characteristics of the population of the study and cohort classification

We included a cohort (C1) of 54 pediatric patients diagnosed with MB, with a male-to-female ratio of 1.16:1. The majority of these patients were diagnosed under 10 years of age with a median age of 7 years (range 1-16 y; see Supplementary Table 1). Patients were classified according to lncRNA’s expression and clinical data, accurately reflecting the established molecular consensus groups (Supplementary Figure 2). A summary of the available clinical data of the publicly available independent cohort (C2) can be found in Supplementary Table 1. A diagram showing the flow of patients through the study can be found in Supplementary Figure 3.

### Differential lncRNA expression analysis of the 4 stablished MB groups

Differential expression analysis identified 2,149 deregulated lncRNAs in WNT MBs, while in the SHH group, 4,262 lncRNAs were deregulated in C1. The complete list of deregulated lncRNAs is provided in Supplementary Table 2. Table 1 shows the log2FC in the top 5 upregulated and downregulated lncRNAs for each group. We observed a 62% and 49% overlap of expression profiles in C2, with all the top upregulated and downregulated genes present in the dataset (Supplementary Table 3). For the non-WNT/SHH groups, 4,202 and 6,264 differentially expressed lncRNAs were identified in Gr3 and Gr4 MBs of C1, respectively. Table 2 shows the log2FC in the top 5 upregulated and downregulated lncRNAs for Gr3 and Gr4 groups. We found a 43% and 63% overlap of lncRNAs in C2, with the majority of the top upregulated and downregulated genes present in the dataset (Supplementary Table 3).

**Table 1.** Top 5 upregulated and downregulated lncRNAs in WNT and SHH groups.

Gene symbol	Log2FC	FDR *P*-value
**WNT**		
NONHSAG086472.1	11.28	0.0003
NONHSAG086471.1	10.77	0.0006
lnckb.107006	10.51	2.5E−13
LOC102723803	10.22	1.8E−14
NONHSAG100968.1	10.20	0.005
NONHSAG100558.1	−27.48	2.1E−35
lnc-RALYL-5	−27.04	3.7E−33
NONHSAG100559.1	−25.92	3.1E−23
NONHSAG100557.1	−25.91	1.1E−29
NONHSAG100568.1	−25.80	5.3E−24
**SHH**		
lnc-BRF1-6	10.01	0.00007
RP11-731F5.1	9.84	3.4E−15
lnckb.113335	8.83	0.005
NONHSAG104540.1	8.80	0.00005
NONHSAG082581.1	8.19	1.3E−11
lnc-BORA-19	−25.61	8.7E−47
LOC105374144	−24.75	2.2E−35
NONHSAG084812.1	−24.62	2E−41
LOC102724419	−24.53	1.8E−36
LOC105377261	−23.86	7E−26

**Table 2.** Top 5 upregulated and downregulated lncRNAs in Gr3 and Gr4 groups.

**Gene symbol**	**Log2FC**	**FDR *P*-value**
**Gr3**		
lnckb.101910	10.04	2.3E−09
LOC105370253	9.53	2.3E−10
NONHSAG067591.2	9.42	5.7E−13
lnckb.101906	9.39	8.9E−09
lnckb.101913	9.37	2.1E−13
DKFZp434L192	−24.18	6.9E−44
AC067960.1	−21.63	6E−23
LINC01419	−9.98	9.8E−16
NONHSAG086472.1	−9.43	0.005
lnckb.110260	−8.48	0.0008
**Gr4**		
lnckb.110263	8.99	2E−09
lnckb.110260	8.85	2E−07
lnckb.110262	8.67	2E−09
lnc-SEMA3A-1	8.58	2.4E−07
lnckb.110261	8.34	2.6E−10
RP11-21B23.3	−24.81	8.7E−44
CATG00000017761.1	-23	7E−13
LOC105370287	−10.26	0.0001
lnc-BRF1-6	−9.84	0.0002
NONHSAG086472.1	−9.5	2.8E−06

### Construction of the coexpression network and identification of group-specific modules

**Figure 1.**
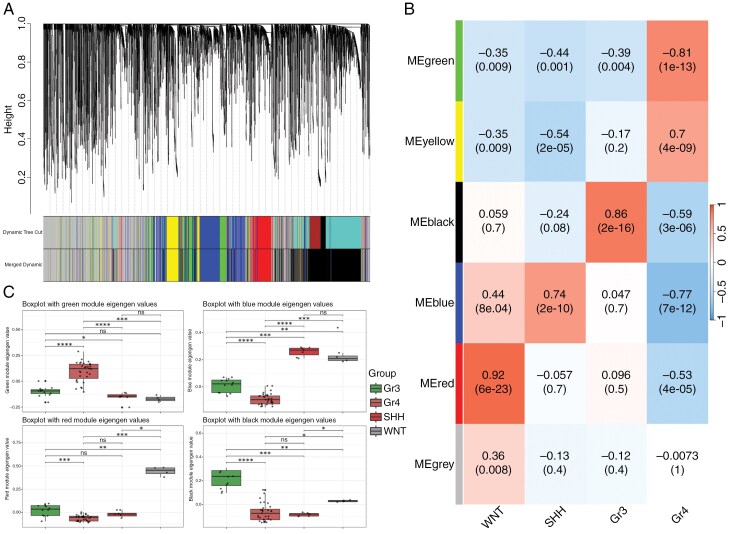
A) Dendrogram illustrating the correlation of genes within coexpression modules identified by WGCNA in C1, with genes coexpressed within the same module represented by a shared color; B) module-trait association results based on the eigengene statistical values (*r*- and *P*-values) between each module and trait; C) boxplots of module’s eigengene values showing the global expression and statistical significance of the selected modules with each trait in C1.

WGCNA resulted in 6 coexpression modules ranging from 325 to 1,830 genes (Figure 1A and Supplementary Table 4). Module–trait associations were estimated using module eigengene values, considering the 4 molecular groups as traits. As shown in Figure 1B, 4 modules were selected for further analyses: the red module, significantly correlated with WNT-MB (*r* = 0.92, *P* = 6 × 10⁻²³); the blue module, with SHH MB (*r* = 0.74, *P* = 2 × 10⁻¹⁰); the black module, with Gr3 MB (*r* = 0.86, *P* = 2 × 10⁻¹⁶); and the green module, correlated with Gr4 MB (*r* = 0.81, *P* = 1 × 10⁻¹³) (Figure 1B and Supplementary Table 4). Boxplots of eigengene values for the 4 selected modules across the consensus molecular groups confirmed the expression patterns and statistical significance of each module’s association with its respective group (Figure 1C and Supplementary Table 4).

### Overrepresentation analyses of group-specific module–trait associations

**Figure 2.**
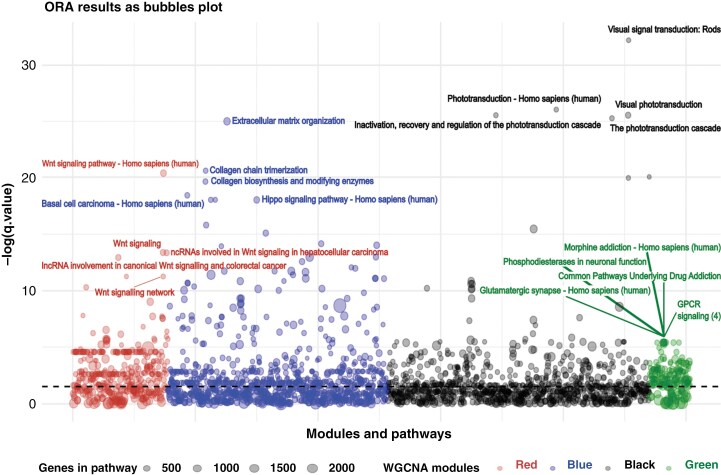
Overrepresentation results of the 4 selected significant modules in ConsensusPathDB as bubble plot. The top 5 most significant pathways with the highest effective size are represented.

Overrepresentation analyses of each module revealed that genes within the WNT MB-correlated red module displayed significant enrichment in 142 pathways, with WNT signaling as the most prominent. For the SHH-associated blue module, 173 pathways showed significant results, with extracellular matrix organization, collagen formation, and Hippo signaling pathways being the most notable, as expected. Modules associated with Gr3 and Gr4 MBs showed enrichment in 162 and 8 pathways, respectively, with pathways characteristic of phototransduction cascade and GPCR signaling highly representative of these groups (Figure 2 and Supplementary Table 5).

### Identification of the group-specific lncRNA–protein–coding gene interactions and hub nodes

**Figure 3.**
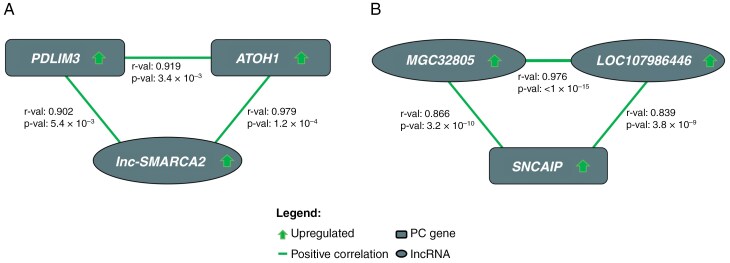
Group-specific lncRNA–protein–coding gene interaction networks for A) SHH MB and B) Gr4 MB. Ellipses indicate lncRNAs, while rectangles represent protein-coding genes. All genes were upregulated in SHH or Gr4 groups respectively, showing positive correlations between the lncRNA-PC gene pairs.

Correlation analyses identified 7,064 significant lncRNA–protein–coding gene interactions in C1 within the blue module, which was associated with the SHH group (Supplementary Table 6). Of these, 77 interactions (50%) were confirmed independently out of the 154 discovered in C2, involving 39 protein-coding genes and 39 lncRNAs (Supplementary Table 7). Among these interactions, the SHH-upregulated lncRNA *lnc-SMARCA2* (*LINCR-0003*) emerged as a hub gene strongly correlated with *PDLIM3* (*r* = 0.902, *P* = 5.4 × 10⁻³) and TF *ATOH1* (*r* = 0.979, *P* = 1.2 × 10⁻⁴), both of which were upregulated in SHH-MBs. Additionally, *PDLIM3* and *ATOH1* exhibited a strong correlation with each other (*r* = 0.919, *P* = 3.4 × 10⁻³) (Figure 3A).

In the analysis of Gr3 MBs, 8,012 significant interactions between protein-coding genes and lncRNAs were identified in C1 (Supplementary Table 6). Of these, 141 interactions (62%) out of the 228 found in C2 were confirmed, involving 89 protein-coding genes and 106 lncRNAs (Supplementary Table 7). This molecular group showed the highest amount of lncRNAs significantly deregulated and correlated with protein-coding genes. Among them, 8 different interactions included genes overlapping the 5 most significantly overrepresented pathways in this molecular group (Supplementary Table 7). These lncRNA–protein–coding gene interactions included *LRP2*, *RGS9BP*, *GRK1*, *PDE6G*, *SAG*, and *CNGA1*; and the lncRNAs *LOC105377123*, *lnc-ANKRD27-6*, *AP000997.2*, *FAM138E*, and *RP11-503G7.2* (Supplementary Figure 4).

In Gr4 MBs, 130 significant lncRNA–protein–coding gene interactions were identified in C1 (Supplementary Table 6), of which 28 were confirmed in C2, showing a complete reproducibility. These interactions included 16 protein-coding genes and 23 lncRNAs (Supplementary Table 7). Among these, *SNCAIP* was correlated with 2 lncRNAs: *MGC32805* (*r* = 0.866, *P* = 3.2 × 10⁻¹⁰) and *LOC107986446* (*r* = 0.839, *P* = 3.8 × 10⁻⁹). Notably, both lncRNAs, as *SNCAIP*, were upregulated and positively correlated (*r* = 0.976, *P* < 1 × 10⁻¹⁵) (Figure 3B). All the correlations found in C2 for each molecular group can be found in Supplementary Table 8.

### Inferred regulatory mediators of lncRNA–protein–coding gene interactions

Regulatory network analysis revealed 365 gene interactions in SHH patients. The resulting GRN confirmed the interactions between *lnc-SMARCA2* and the *ATOH1* and *PDLIM3* genes identified in correlation analyses, exhibiting notably high mutual information values (Supplementary Table 9). Furthermore, strong interactions were observed between TFs *ESR2*, *SATB1*, *ZNF121*, and *ZNF662*, and the candidate *lnc-SMARCA2* (Supplementary Table 9).

In Gr4 MBs, 349 interactions were identified between regulatory genes and the candidate genes previously identified by correlation analysis. Consistent with our findings, correlations between lncRNAs *MGC32805* and *LOC107986446* and the *SNCAIP* gene were confirmed in the GRN. Notably, 23 TFs were found to significantly interact with both *MGC32805* and *LOC107986446* lncRNAs (Supplementary Table 9). Among these, *RPH3A*, *TGIF2*, and *TMEM108* were the only TFs significantly differentially expressed in Gr4 patients (Supplementary Table 2).

## Discussion

The interest in the role of lncRNAs in MB has increased in recent years as they may play key roles in the disease, displaying neuro-anatomical and/or cell-type specific expression and functions.^34^ Consequently, the lncRNA landscape may clarify the characterization of the heterogeneity of the molecular groups which have the lowest OS rates.^35^ Here, the transcriptomic characterization of an MB cohort confirmed lncRNAs as effective biomarkers for molecular group classification. This characterization revealed 2 critical lncRNA–protein–coding gene interactions, involving 3 lncRNAs, with potential direct implications in the tumorigenicity of SHH and Gr4 MBs, suggesting their potential as therapeutic biomarkers for these molecular groups. Additionally, characterization of the Gr3 molecular group provided insights into the role of lncRNAs in shaping the tumor phenotype in these malignancies.

Our study represents one of the few genome-wide analyses of lncRNA expression and biomarker potential across MB molecular groups. Hierarchical clustering based on highly variable lncRNAs revealed 4 distinct expression clusters, likely overlapping with the known molecular groups, suggesting that lncRNAs may contribute to group-specific tumor biology.^36^

Differential expression analysis between MB groups revealed group-specific lncRNA deregulation patterns. Notably, the majority of the most highly expressed and deregulated lncRNAs were detected across cohorts. However, many of the top upregulated and downregulated lncRNAs correspond to uncharacterized RNA genes, highlighting the need for further functional characterization of lncRNAs. Regarding previously reported lncRNAs *HHIP-AS1* and *LOXL1-AS1*, DEA showed differential expression in SHH group as expected, while tumor suppressor lncRNA *NKX2-2AS* was upregulated in this group.^13–15^*LINC-NED125*, *CRNDE*, and *OTX2-AS1*, which affect tumor progression in MB, showed differential expression patterns between groups.^18,20,21^ Additionally, *CCAT1* and *USP2-AS1* were found upregulated, while *MIR100HG* was downregulated in Gr3 MBs, consistent with prior findings.^36^ The expression profiles observed in our study not only confirmed the relevance of several previously characterized oncogenic and tumor-suppressive lncRNAs but also revealed novel group-specific lncRNAs which could be key molecular group biomarkers of MB.

To investigate the oncogenic specificity of lncRNAs in each of the molecular groups, WGCNA was carried out in C1. WGCNA led to the selection of 4 modules of coexpressed genes showing high correlation with WNT, SHH, Gr3, and Gr4 MBs. Given the limited understanding of lncRNA function, correlation analyses between lncRNAs and protein-coding of each group were performed in C1 and tested in C2 to infer the possible role of the lncRNAs in MB.

LncRNA-protein-coding gene correlation analysis detected 2 potentially key interactions in SHH and Gr4 MBs involving 3 different lncRNAs and driver SHH and Gr4 MB genes. Analysis of SHH MB revealed a critical interaction involving the 2 SHH driver genes, *ATOH1* and *PDLIM3*, with *lnc-SMARCA2*. Intriguingly, these protein-coding genes were included by Northcott PA et al. in the group-specific 22-gene signature used to proxy our cohort classification.^37^ Sonic hedgehog signaling activation is crucial for the normal development of the cerebellar granule neuron progenitors (GNPs), cell of origin of SHH-activated MBs. Granule neuron progenitor cell proliferation is driven by the activation of the SHH signaling cascade, a process exclusively occurring in primary cilia.^38^*ATOH1* and *PDLIM3* are master regulators of primary cilia formation and maintenance in GNPs, with the TF *ATOH1* maintaining primary cilia in GNPs, ensuring the cells’ sensitivity to SHH protein even as they mature,^39^ and *PDLIM3* functioning as a regulator of cilia formation, activating SHH signaling through membrane cholesterol tethering.^40^ Given their critical roles in SHH MB development, our findings suggest that *lnc-SMARCA2* may play a crucial regulatory role in the TF *ATOH1* potentially modulating *PDLIM3* expression, contributing to the cilium-mediated deregulation of SHH signaling. Indeed, *lnc-SMARCA2* has been previously implicated in liver cancer as a regulator of the SWI/SNF chromatin modulator complex. By interacting with the SMARCA2 subunit, it induces an SMARCA2/SMARCA4 switch, constitutively activating *SMARCA4* and deregulating its downstream targets.^41^ Notably, *ATOH1* has been identified to be coordinated with *SMARCA4* to regulate specific MB oncogenes.^42^ All together, these results present a complex regulatory network involving *ATOH1*, *PDLIM3*, and *lnc-SMARCA2* that could mediate SHH MB development. Overexpression of *lnc-SMARCA2* detected in SHH patients may lead to aberrant expression of *ATOH1* and *PDLIM3* genes, facilitating cilium formation and maintenance constitutively activating SHH signaling. Moreover, deregulation of *ATOH1* through *lnc-SMARCA2* overexpression and the known *SMARCA4* activation by this lncRNA, may enhance the *ATOH1*-*SMARCA4* interactions involving regulation of potent MB oncogenes.

In order to explore potential regulatory interactions of lncRNAs and considering the fact that lncRNAs can recruit and modulate the DNA binding activity of TFs,^43^ GRN analysis was performed to infer other potential TFs interacting with candidate lncRNAs. In SHH MB patients, the GRN analysis revealed 5 TFs interacting with *lnc-SMARCA2*: *ATOH1*, *ESR2*, *SATB1*, *ZNF121*, and *ZNF662*. Among them, *ATOH1* was the only 1 deregulated and associated with MB disease. Moreover, *ATOH1* was also found to interact with *PDLIM3*, confirming the *lnc-SMARCA2*-*ATOH1*-*PDLIM3* GRN axis proposed before through WGCNA. These findings support the hypothesis that *lnc-SMARCA2* upregulates the expression and activity of the TF *ATOH1*, potentially promoting *PDLIM3* upregulation and forming a positive feedback loop contributing to SHH MB pathogenesis through cilium-related mechanisms. Although further functional validation is needed, *lnc-SMARCA2* emerges as a potential biomarker and transcriptional regulator in SHH MB, with promising implications for targeted therapeutic strategies.

Regarding Gr4 group, correlation analyses revealed a critical interaction network involving lncRNAs *MGC32805* and *LOC107986446*, and *SNCAIP* gene. *SNCAIP*, a known driver gene in Gr4 MB, involved in neurodegenerative pathways as revealed in ORA, is frequently targeted by recurrent tandem duplications, affecting ~17% of patients within this group.^1^ Notably, the *SNCAIP* locus overlaps with a Gr4-specific superenhancer region.^44^ Tandem duplication of this region is hypothesized to activate the expression of the *PRDM6* gene, located ~600 kb downstream of the *SNCAIP* locus, by disrupting the local chromatin architecture and forming novel chromatin loops that facilitate enhancer–promoter interactions.^7^ The upregulation of *PRDM6* is considered pivotal in Gr4 MB pathogenesis, supported by recent evidence, including findings from our cohort, showing that *PRDM6* overexpression promotes MB development.^45^ On the other hand, the lncRNAs *MGC32805* and *LOC107986446*, located overlapped ~30 kb downstream of the *SNCAIP* locus, are both highly expressed in brain tissues. While *MGC32805* has been previously associated with overexpression in high-grade glioma,^46^ no definitive oncogenic roles have been reported for these lncRNAs in brain tumors. Nonetheless, their location within the *SNCAIP* super-enhancer region suggests a potential role in the activation of *PRDM6* through enhancer hijacking, a process potentially facilitated by the intrinsic properties of *cis*-acting lncRNAs. Importantly, one of their primary functions involves regulating enhancer activity by maintaining or forming chromatin loops that promote interactions between DNA binding proteins, enhancers, and target gene promoters.^47^ Given their location within the duplicated *SNCAIP* super-enhancer regions in Gr4 patients, it is plausible that *MGC32805* and *LOC107986446* are duplicated along with *SNCAIP*, contributing to the formation and maintenance of chromatin loops necessary for interaction between the super-enhancer and the *PRDM6* promoter. Such mechanism may support constitutive activation of *PRDM6*, driving Gr4 MB tumorigenesis.

Following these findings, GRN analysis was performed in Gr4 patients to explore potential TFs involving lncRNA-mediated *PRDM6* activation. Although no direct interactions between the candidate lncRNAs and *SNCAIP* were observed, GRN analysis revealed *RPH3A*, *TGIF2*, and *TMEM108* as the most relevant TFs interacting with lncRNAs *MGC32805* and *LOC107986446*. Notably, *RPH3A* gene, which encodes an effector of small G-proteins involved in the late stages of neurotransmitter exocytosis, has been previously reported upregulated in Gr4 MB at both the transcriptional and protein level.^48^ Therefore, considering the combined mechanism of actions of lncRNAs involving the formation of chromatin loops and recruitment of regulatory TFs,^43^ it is conceivable that *MGC32805* and *LOC107986446* recruit TFs such as *RPH3A* to enhance and constitutively activate *PRDM6* expression. These findings propose *MGC32805* and *LOC107986446* as potential candidate biomarkers for Gr4 MB that should be tested for further functional studies to elucidate their precise roles in *PRDM6* activation and Gr4 pathogenesis.

Finally, Gr3 MB exhibited the highest representation of lncRNAs interactions suggesting a pivotal role for lncRNAs in the deregulation of pathways implicated in Gr3 development. Despite this, no driver genes were identified within the correlations, hindering the identification of Gr3-specific lncRNA biomarkers. However, 5 lncRNAs—*LOC105377123*, *lnc-ANKRD27-6*, *AP000997.2*, *FAM138E*, and *RP11-503G7.2*—were found strongly correlated with 6 genes involved in the visual phototransduction cascade, as indicated in the ORA. Recent studies have shown that Gr3 MB tumors adopt retinal photoreceptor lineage identity driven by dysregulation of visual transduction cascade-related pathway.^49^ While further investigation is required to elucidate the precise role of these lncRNAs in Gr3 MB development, our analysis revealed that the validated correlations between the 6 phototransduction-related genes and the 5 lncRNA signature may reflect a complex regulatory network linking these lncRNAs to the switch between normal upper rhombic lip-derived unipolar brush cells and the photoreceptor lineage in Gr3 MB.

Although this study provides a comprehensive transcriptomic characterization of the lncRNA landscape across MB molecular groups, certain limitations should be acknowledged. The limited sample size of the discovery cohort (C1) precluded an exhaustive analysis of the WNT group in correlation analyses. Similarly, while the methodological approach allowed for the analysis of Gr3 MB, the relatively small sample size may have hindered the detection of potentially direct oncogenic lncRNA–protein–coding gene interactions. Nonetheless, the inclusion of an independent cohort comprising 207 patients helps mitigate random variation, thereby strengthening the reliability of the findings. Finally, although C2 may be less sensitive for detecting associations due to its heterogeneous composition, the cross-cohort validation highlights the most robust and biologically consistent gene correlations, which are reproducible across very different contexts.

Overall, our study identifies *lnc-SMARCA2*, and *MGC32805* and *LOC107986446* as potential group-specific lncRNA biomarkers for SHH and Gr4 MB, respectively, highlighting their potential role as therapeutic biomarkers, given their interactions with these molecular group`s driver oncogenes, and potential direct involvement in disease pathogenesis. While these findings require further research and validation to confirm their clinical relevance and spread this search to WNT and Gr3 groups, this work offers new perspectives into the role of lncRNAs in MB pathogenesis. Due to the lack of studies involving lncRNA and MB development, this study provides novel insights and may be the starting point for further research involving these emerging biomarkers which could lead to important breakthroughs in MB pathogenesis.

## Supplementary Material

vdaf194_suppl_Supplementary_Tables_1

vdaf194_suppl_Supplementary_Tables_2

vdaf194_suppl_Supplementary_Tables_3

vdaf194_suppl_Supplementary_Tables_4

vdaf194_suppl_Supplementary_Tables_5

vdaf194_suppl_Supplementary_Tables_6

vdaf194_suppl_Supplementary_Tables_7

vdaf194_suppl_Supplementary_Tables_8

vdaf194_suppl_Supplementary_Tables_9

vdaf194_suppl_Supplementary_Figures_1

vdaf194_suppl_Supplementary_Figures_2

vdaf194_suppl_Supplementary_Figures_3

vdaf194_suppl_Supplementary_Figures_4

## Data Availability

All data generated or analyzed during this study are included in this manuscript (and its Supplementary material files). RNA-sequencing data are available at the Sequence Read Archive (SRA) upon publication under accession number PRJNA1186080 (https://dataview.ncbi.nlm.nih.gov/object/PRJNA1186080?reviewer=q72k6fnmh7vrcf3vqlff44hokm).
