# Supplementary figures and images for: Global lncRNA expression profiles in medulloblastoma reveal crucial lncRNA-oncogene interactions in Sonic hedgehog and Group 4
Source: Neurooncol Adv. 2025 Sep 4;7(1):vdaf194. doi: 10.1093/noajnl/vdaf194 (PMC12559851; doi:10.1093/noajnl/vdaf194)

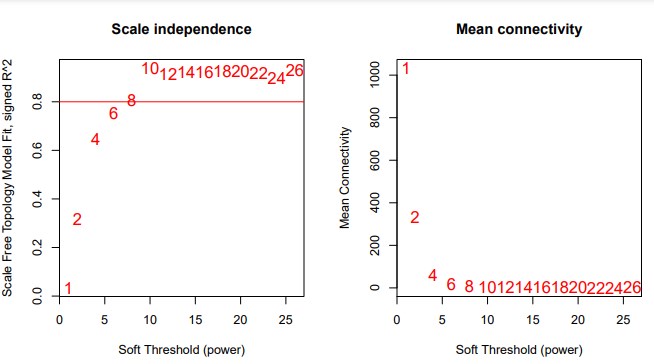

Supplement: vdaf194_suppl_Supplementary_Figures_1 [file vdaf194_suppl_supplementary_figures_1.jpeg]

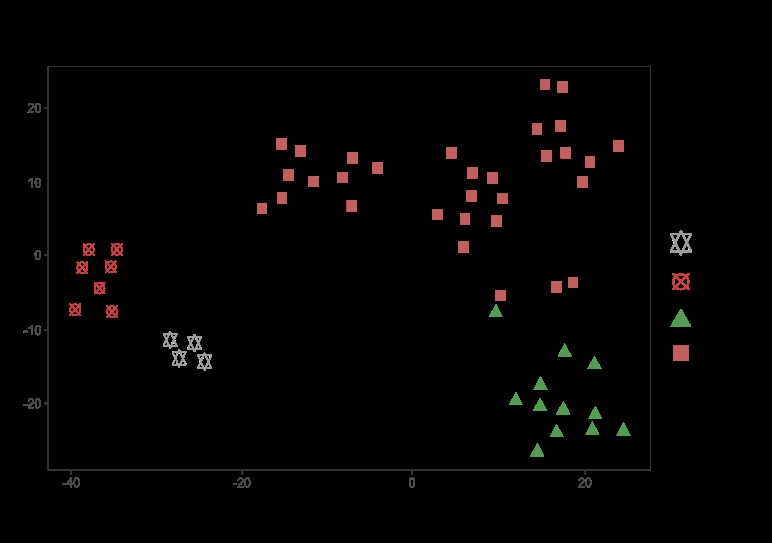

Supplement: vdaf194_suppl_Supplementary_Figures_2 [file vdaf194_suppl_supplementary_figures_2.jpeg]

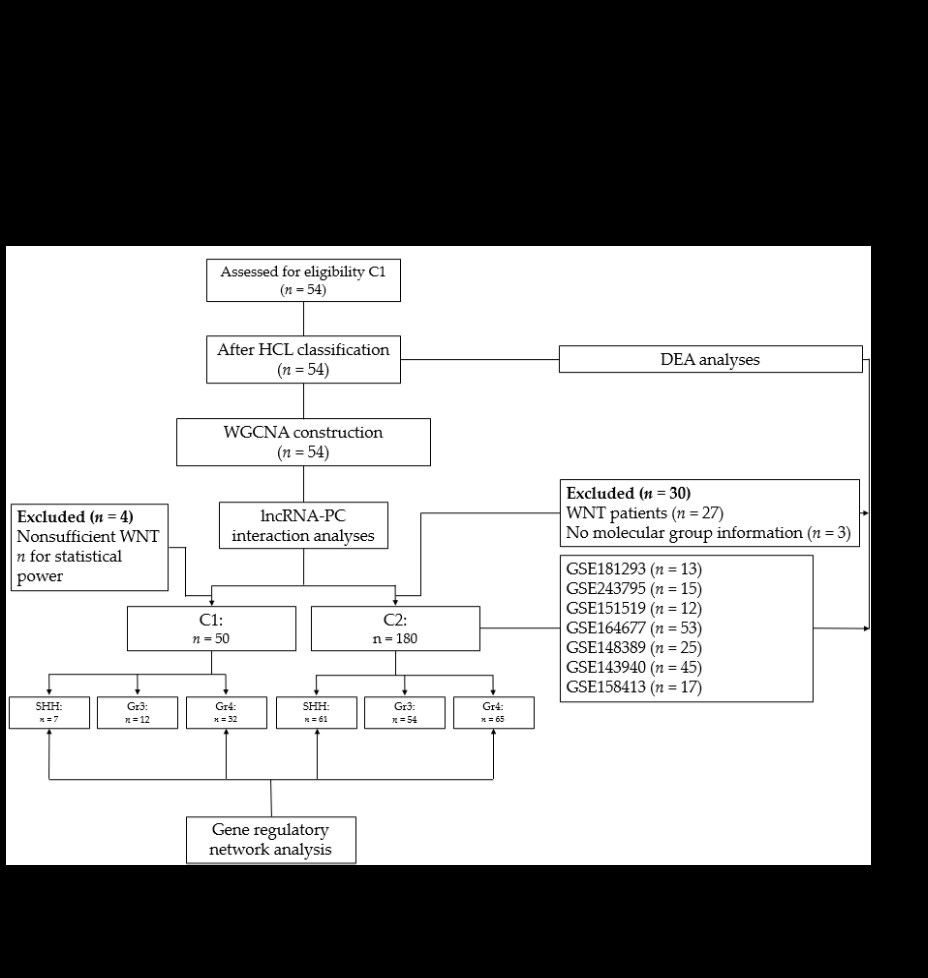

Supplement: vdaf194_suppl_Supplementary_Figures_3 [file vdaf194_suppl_supplementary_figures_3.jpeg]

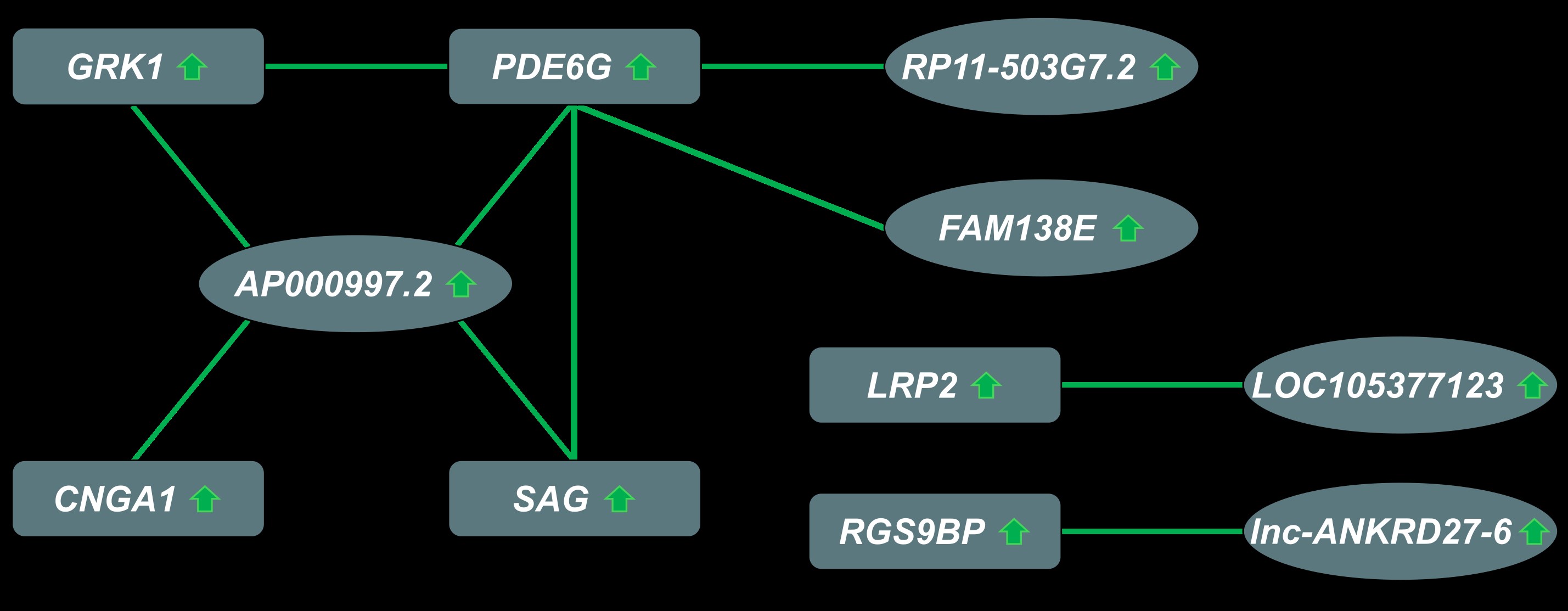

Supplement: vdaf194_suppl_Supplementary_Figures_4 [file vdaf194_suppl_supplementary_figures_4.jpeg]
